# Infection of *Helicobacter pylori* and Atrophic Gastritis Influence *Lactobacillus* in Gut Microbiota in a Japanese Population

**DOI:** 10.3389/fimmu.2018.00712

**Published:** 2018-04-06

**Authors:** Chikara Iino, Tadashi Shimoyama, Daisuke Chinda, Tetsu Arai, Daisuke Chiba, Shigeyuki Nakaji, Shinsaku Fukuda

**Affiliations:** ^1^Department of Gastroenterology and Hematology, Hirosaki University Graduate School of Medicine, Hirosaki, Japan; ^2^Department of Gastroenterology and Hematology, Hirosaki National Hospital, Hirosaki, Japan; ^3^Department of Social Medicine, Hirosaki University Graduate School of Medicine, Hirosaki, Japan

**Keywords:** *Lactobacillus*, *Helicobacter pylori*, atrophic gastritis, gut microbiota, pepsinogen

## Abstract

**Background:**

Suppression of gastric acid by proton pump inhibitors is associated with the increase of *Lactobacillus* in human gut microbiota. Gastric acid secretion is also suppressed by *Helicobacter pylori* infection and following atrophic gastritis. However, few studies have examined the association between *H. pylori* infection and *Lactobacillus* species in gut microbiota particularly in Japan.

**Methods:**

A total of 1,123 adult subjects who participated in a health survey in Hirosaki City were studied. Infection of *H. pylori* was defined by both serum antibody and stool antigen test. The presence and the severity of atrophic gastritis were defined by the serum level of serum pepsinogens. Using 16S ribosomal RNA amplification from fecal samples, the relative abundance of *Lactobacillus* was calculated, and the composition ratio of each *Lactobacillus species* was surveyed.

**Results:**

The relative abundance of the *Lactobacillus* in *H. pylori*-infected subjects with severe atrophic gastritis was higher comparing with those in subjects with mild atrophic gastritis and without atrophic gastritis (0.591 vs 0.068% and 0.033%, respectively; *p* < 0.001) and also that of non-infected subjects (0.033%; *p* < 0.001). In *H. pylori* non-infected subjects, both gender and age were not associated with the relative abundance of *Lactobacillus* in fecal samples. The proportion of *Lactobacillus salivarius* was high in *H. pylori*-infected subjects while that of *Lactobacillus acidophilus* was high in non-infected subjects.

**Conclusion:**

*Lactobacillus* in human gut microbiota could be influenced by *H. pylori* infection and severity of atrophic gastritis in Japanese subjects.

## Introduction

*Lactobacilli* are a well-known probiotic and have been introduced into many fermented dairy products. A recent meta-analysis of randomized controlled trials showed products containing *Lactobacillus* species increased stool frequency in constipated adults (1). Several randomized controlled studies also reported that some *Lactobacillus* species such as *Lactobacillus acidophilus, Lactobacillus bulgaricus, Lactobacillus casei*, and *Lactobacillus rhamnosus* GG prevented antibiotic-associated diarrhea and *Clostridium difficile*-associated colitis (2–5).

Recent studies suggested that the use of proton pump inhibitors (PPIs) was associated with the increase in the *Lactobacillus* population in the human gut microbiota (6, 7). This phenomenon is thought to be due to long-term acid suppression by PPIs. Gastric acid secretion is also suppressed by *Helicobacter pylori* infection and following atrophic gastritis (8–12). However, it is unclear whether the decrease of gastric acid caused by *H. pylori* infection and atrophic gastritis would cause an increase in *Lactobacillus* in the human gut microbiota. Most patients infected with *H. pylori* develop atrophic gastritis in Japan (13). Gastric acid secretion is also lower in Japanese people comparing with Western populations in healthy subjects (14). The lower gastric acid secretion might result in different gut microbiota in Japanese from those in Western people. Indeed, a recent study demonstrated that gut microbiome of the Japanese is considerably different from those of other populations (15). In Japan, however, few studies have examined the association between *H. pylori* infection and gut microbiota, particularly *Lactobacillus* species. Although a previous German study showed a modulation of *Lactobacillus* in the gut microbiota after successful eradication of *H. pylori*, next-generation sequence analysis was not used (16). Furthermore, previous studies have been performed regardless of the extent of atrophic gastritis.

The aim of this study was to evaluate the influence of *H. pylori* infection and the progress of atrophic gastritis on the amount and diversity of *Lactobacillus* species in the human gut microbiota in Japan using next-generation sequence analysis.

## Materials and Methods

### Study Subjects

**Figure 1:**
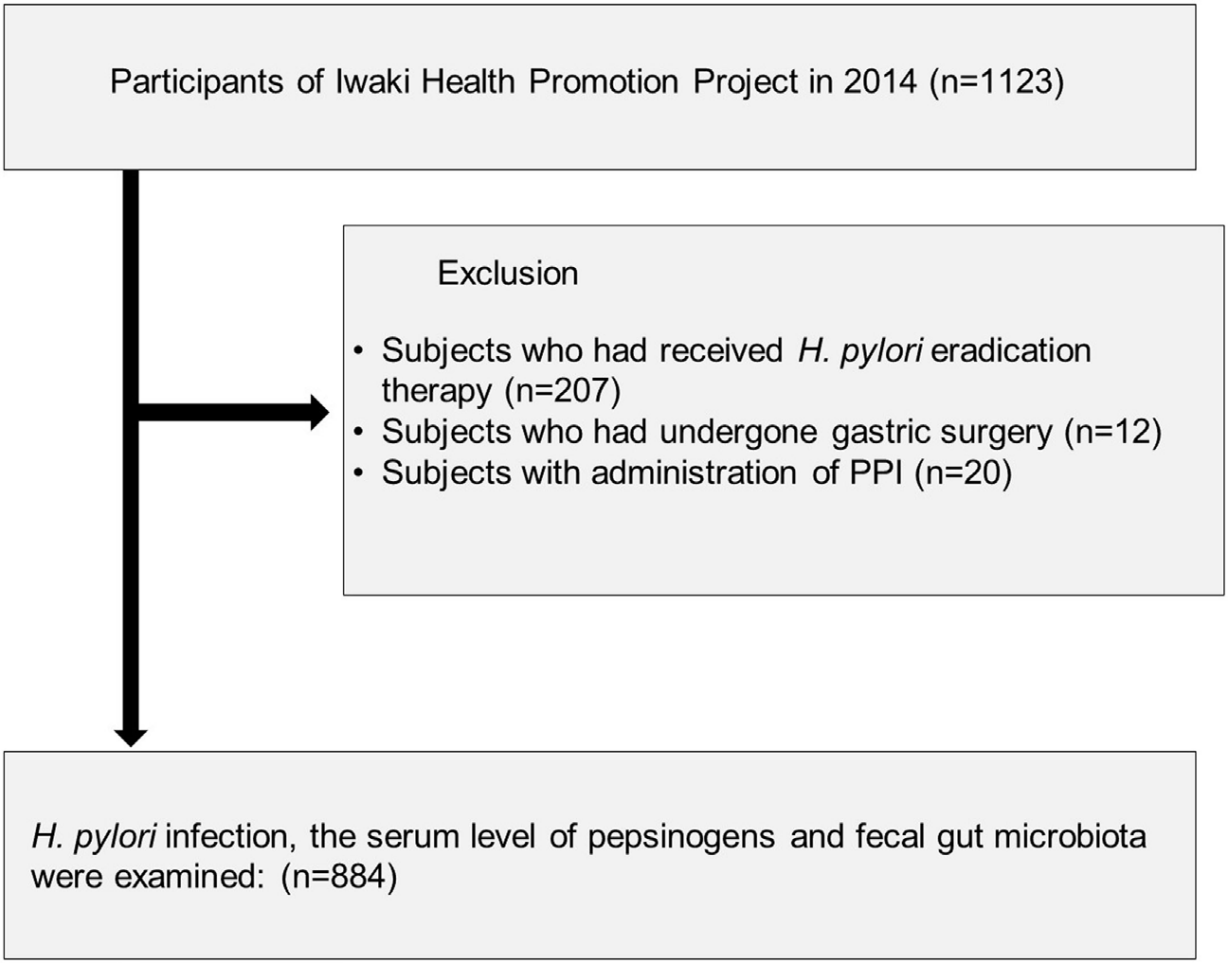
Study flow of subjects. A total of 884 subjects were enrolled from 1,123 adults who participated in the Iwaki Health Promotion Projects in 2014.

A total of 1,123 adults participated in the Iwaki Health Promotion Projects held in June 2014, in Hirosaki City, north Japan (Figure 1). Of these, we excluded 207 subjects who had previously received *H. pylori* eradication therapy, 12 subjects who had a previous history of gastric surgery, and 20 subjects who were taking PPI. After the exclusion, there was no subject whose serum level of creatinine was larger than 2.0 mg/dL. Finally, 884 subjects were analyzed.

### Diagnosis of *H. pylori* Infection

Serum samples were collected after one night of fasting and stored at −20°C. The titer of serum IgG antibody to *H. pylori* was measured by E-plate (Eiken, Tokyo, Japan) (17). Stool samples were collected and stored at −80°C. Stool samples were tested for *H. pylori* antigen by using Testmate EIA (Wakamoto and Kyowa Medex, Tokyo, Japan) (18). *H. pylori* status was defined as positive when the stool antigen test was positive and serum antibody titer ≥10 U/mL, and as negative when the stool antigen test was negative and serum antibody titer <3 U/mL.

### Evaluation of Atrophic Gastritis

The serum level of pepsinogen (PG) I and II was measured and used as markers of atrophic gastritis (12, 19, 20). The result was considered indicative of atrophic gastritis when both a PG I level of <70 μg/L and a PG I/II ratio of <3.0 were observed, and severe atrophic gastritis when both a PG I level of <30 μg/L and a PG I/II ratio <2.0 were observed.

### DNA Extraction From Fecal Samples

Two to three grams of fecal samples were collected by each participant in commercial containers (TechnoSuruga Laboratory Co., Ltd., Shizuoka, Japan) and suspended in guanidine thiocyanate solution [100 mM Tris–HCl (pH 9.0), 40 mM Tris–EDTA (pH 8.0), 4 M guanidine thiocyanate]. These samples were kept at −80°C until DNA extraction. Frozen fecal solids were beaten with zirconia beads at 5 m/s for 2 min by using a FastPrep 24 Instrument (MP Biomedicals, Santana Ana, CA, USA). DNA was extracted from 200 µL of the suspension by using a Magtration System 12 GC (Precision System Science, Japan), with MagdDEA DNA 200 (Precision System Science, Japan) as the reagent for automatic nucleic acid extraction.

### Next-Generation Sequence Analysis and 16S rDNA-Based Taxonomic Analysis

According to previous studies, a series of representative bacteria in the human gut microbiota was analyzed using the primers for the V3–V4 region of 16S rDNA of prokaryotes (21, 22). Sequencing was conducted using an Illumina MiSeq system (Illumina, San Diego, CA, USA). The method for quality filtering the sequences was as follows: only reads that had quality value scores of scores of ≥20 for more than 99% of the sequence were extracted for the analysis. Detection and identification of bacteria from sequences were performed using Metagenome@KIN software (World Fusion Co., Tokyo, Japan) and the TechnoSuruga Lab Microbial Identification database DB-BA 10.0 (TechnoSuruga Laboratory) at 97% sequence similarity.

We compared the relative abundance of *Lactobacillus* in the gut microbiota between *H. pylori*-infected and non-infected subjects and among subjects with different degrees of atrophic gastritis. Relative abundance is the percent composition of reads of *Lactobacillus* relative to the total reads of gut microbiota. The composition ratio of each *Lactobacillus* species was surveyed in each subject. Moreover, to evaluate the influence of gender and age, in *H. pylori* non-infected subjects, we surveyed the relative abundance of *Lactobacillus* in gut microbiota between male and female, and among different age groups by decades.

### Statistical Analysis

Statistical analyses of the clinical data were performed using the Statistical Package for the Social Sciences (SPSS) version 20.0 (SPSS Inc., Chicago, IL, USA). Categorical variables are shown as frequencies and percentages and, continuous variables are shown as the mean with standard deviation or the median with interquartile range. Categorical variables were compared using the chi-square test and continuous variables were compared using the Student’s *t*-test. One-way ANOVA was used for comparing more than two groups. Mann–Whitney *U*-test and Steel–Dwass test were used to compare the abundance of *Lactobacillus*. A *p* value of less than 0.05 was considered significant for all tests.

### Ethics Statement

This study was performed in accordance with the ethical standards of the Declaration of Helsinki and approved by the ethics committee at Hirosaki University Medical Ethics Committee (2014-377). All patients provided written informed consent for this study.

## Results

**Figure 2:**
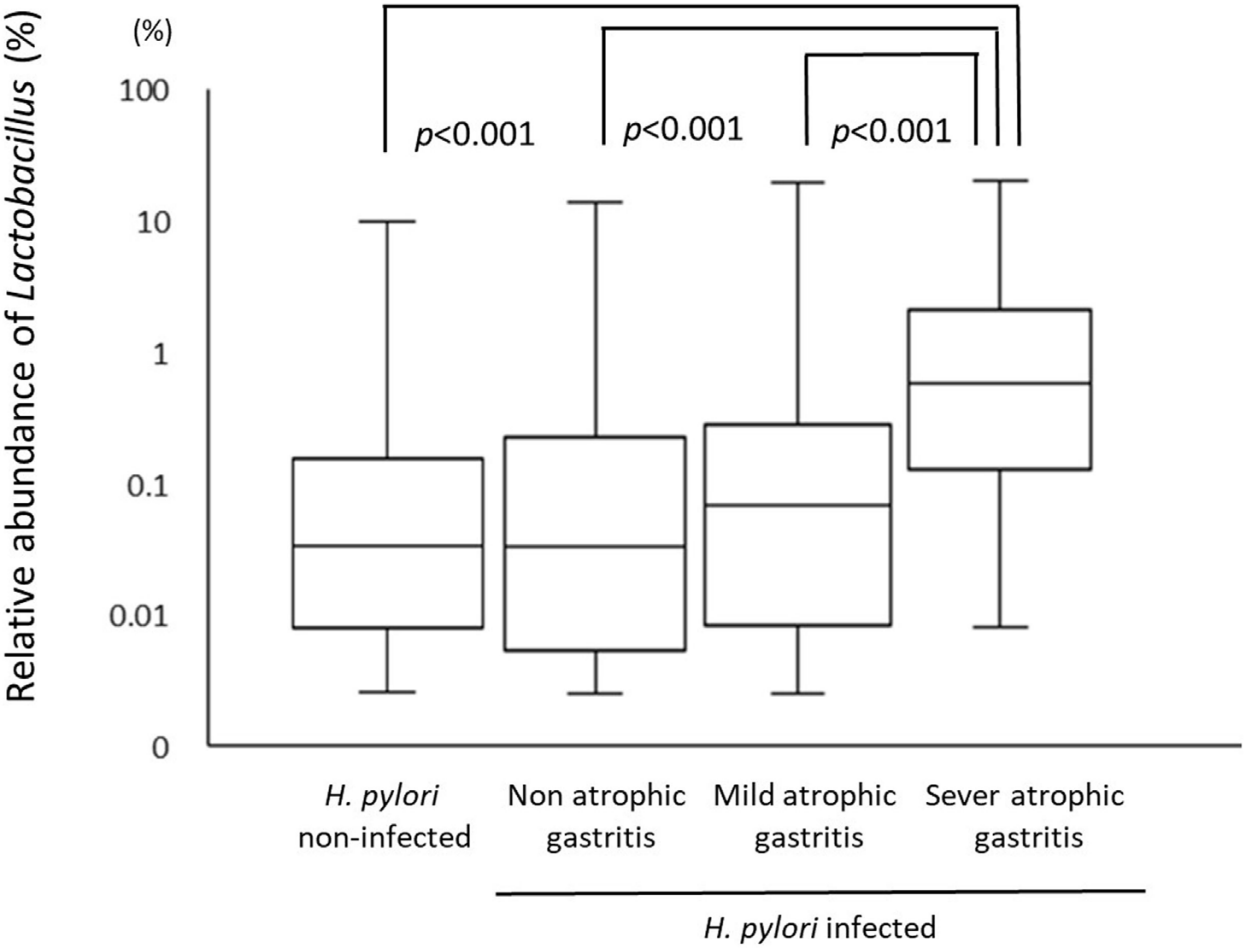
The relative abundance of the *Lactobacillus* in gut microbiota among *Helicobacter pylori* non-infected subjects and infected subjects with different degree of atrophic gastritis.

**Figure 3:**
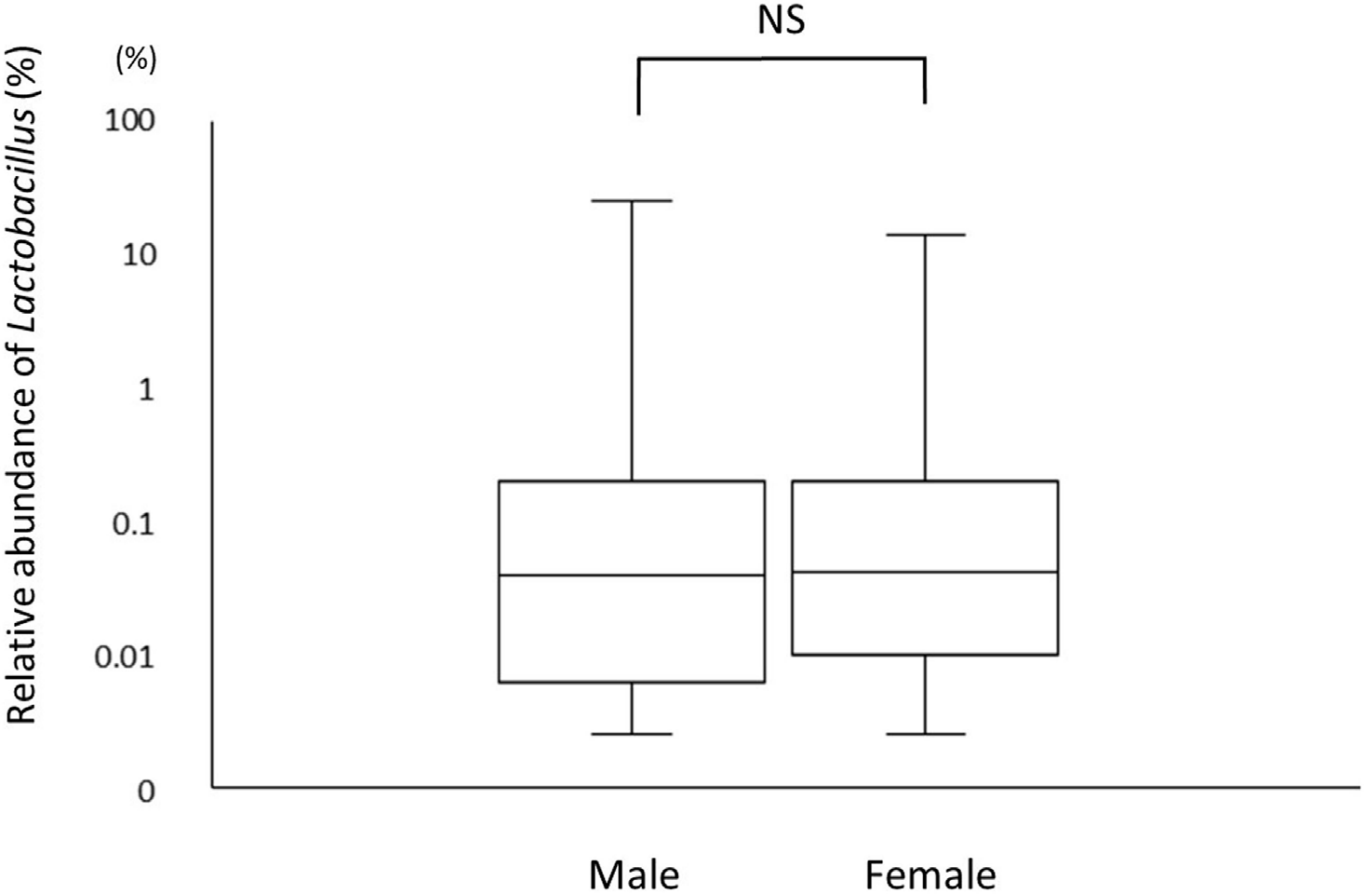
The relative abundances of *Lactobacillus* in gut microbiota in male and female.

**Figure 4:**
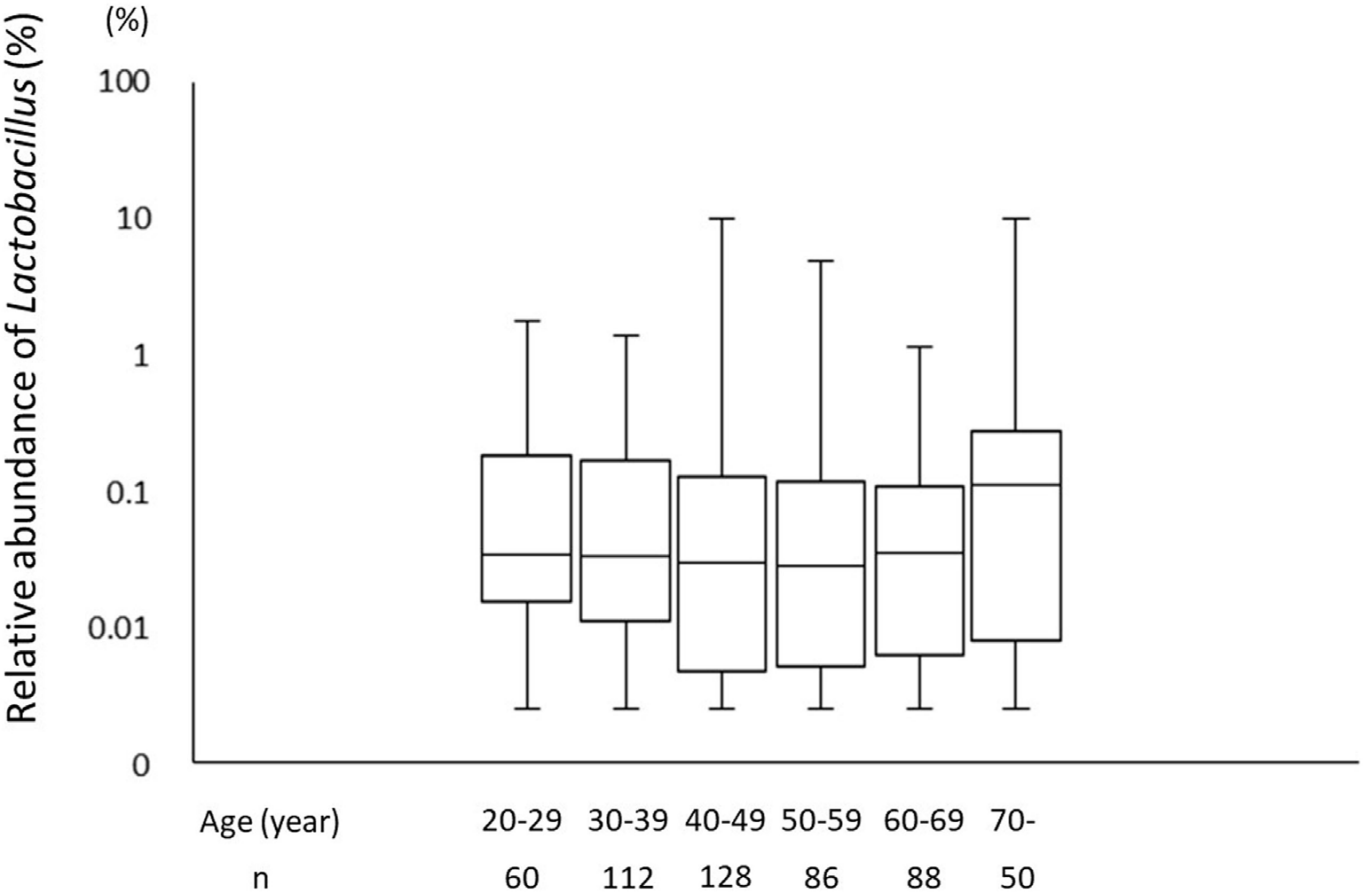
The relative abundance of the *Lactobacillus* in gut microbiota among the six age groups by decades.

A total of 226 subjects were defined as infected with *H. pylori*, and 524 subjects who were defined as non-infected (Table 1). The median relative abundance of *Lactobacillus* in subjects with *H. pylori* infection and non-infected subjects was 0.071 (interquartile range: IQR; 0.350)% and 0.033 (0.143)%. In *H. pylori*-infected subjects, there were 111 subjects without atrophic gastritis, 81 subjects with mild atrophic gastritis and 34 subjects with severe atrophic gastritis (Table 2). The median relative abundance of *Lactobacillus* in subjects with severe atrophic gastritis was significantly higher than those in subjects with mild atrophic gastritis, without atrophic gastritis and non-infected [median (IQR) 0.591 (1.837) vs 0.068 (0.258)%, 0.033 (0.205)% and 0.033 (0.143)%; *p* < 0.001] (Figure 2). The relative abundance of *Lactobacillus* was not significantly different between subjects with mild atrophic gastritis, without atrophic gastritis and non-infected subjects. In *H. pylori* non-infected subjects, there were no significant differences in the relative abundance of *Lactobacillus* between male and female subjects (*p* = 0.332) (Figure 3), or among the six age groups by decades (*p* = 0.532) (Figure 4).

**Table 1:** Subject characteristics according to *Helicobacter pylori* status.

Variable	*H. pylori* status		*p* Value
	Infected	Non-infected	
*n* (%)	226 (30.1)	524 (69.9)	
Male (%)	89 (39.3)	186 (35.4)	0.311
Age, years old (mean ± SD)	59.0 ± 13.6	48.0 ± 15.0	<0.001
Non-existence of *Lactobacillus* (%)	27 (11.9)	71 (13.5)	0.55

**Table 2:** Subject characteristics according to grade of atrophic gastritis.

Variable	Grade of atrophic gastritis			*p* Value
	Non	Mild	Severe	
*n* (%)	111 (49.1)	81 (35.8)	34 (15.1)	
Male (%)	49 (44.1)	24 (29.6)	16 (47.1)	0.077
Age, years old (mean ± SD)	54.1 ± 14.1	61.1 ± 10.9	69.9 ± 9.5	<0.001
Non-existence of *Lactobacillus* (%)	18 (16.2)	9 (11.1)	0 (0)	0.037

**Figure 5:**
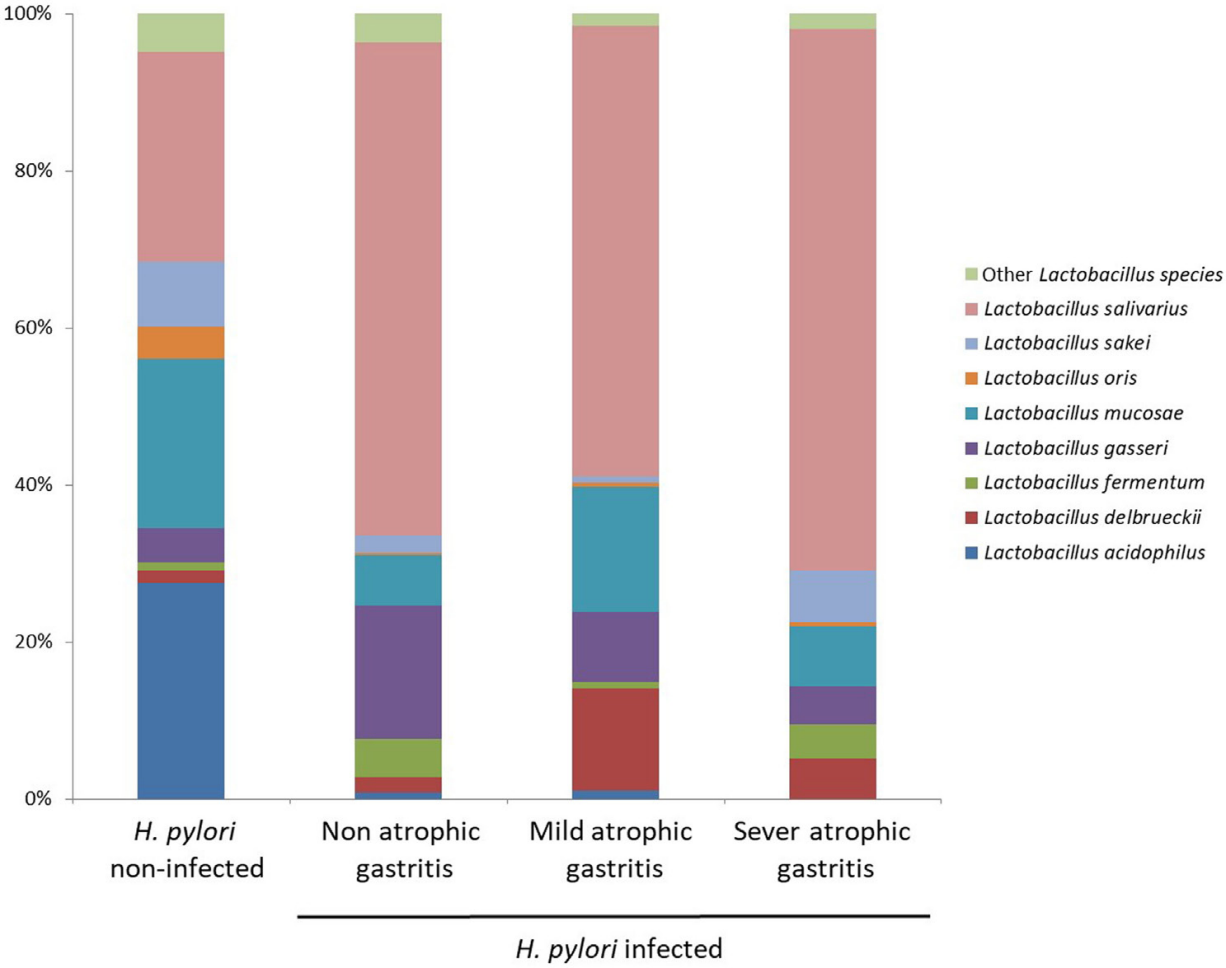
Proportion of *Lactobacillus* species in *Helicobacter pylori*-infected subjects divided by degree of atrophic gastritis and non-infected subjects.

Figure 5 shows the proportion of *Lactobacillus* species in *H. pylori*-infected subjects divided by degree of atrophic gastritis and non-infected subjects. The proportion of *Lactobacillus salivarius* was higher in *H. pylori*-infected subjects than non-infected subjects. By contrast, the proportion of *L. acidophilus* in *H. pylori* non-infected subjects was higher than that in *H. pylori*-infected subjects regardless the degree of atrophic gastritis.

## Discussion

This study demonstrated that infection with *H. pylori* modulates the proportion of species of *Lactobacillus* in gut microbiota. The relative abundance of *Lactobacillus* in human gut microbiota may increase after the development of severe atrophic gastritis. By contrast, in *H. pylori* non-infected subjects, relative abundance of *Lactobacillus* in gut microbiota was not affected by gender or age.

A previous German study reported that the presence of *H. pylori* led to an increased growth of lactobacilli (16). In this study, however, *H. pylori*-infected subjects showed higher rates of *Lactobacillus* only when the subjects had severe atrophic gastritis. Therefore, the rates of *Lactobacillus* in the gut microbiota could be associated with the progress of atrophic gastritis rather than with *H. pylori* infection in Japan. Severe gastric atrophy indicated by serum level of PGs has been significantly correlated with gastric acid secretion (12, 23). The use of PPIs has been associated with an increase of *Lactobacillus* in the gut microbiota (6, 7). In Japan, the reduction of gastric acid by severe atrophic gastritis could influence the rates of *Lactobacillus* in gut microbiota even though the effects were smaller than with the use of PPI or potassium-competitive acid blockers. By contrast, in Western people, *H. pylori*-infected in only gastric antrum to pylorus, so the gastric acid would be increased by *H. pylori* infection. Therefore, species of *Lactobacillus* would be different between Japanese and Western people even though *H. pylori* infection is associated with the increase of *Lactobacillus* in both populations.

Generally, the development of atrophic gastritis in males is observed at a younger age than in females. Furthermore, the degree of atrophic gastritis increases with age, alongside the duration of *H. pylori* infection. Therefore, to eliminate the potential influence of gender or age on our results, we compared the relative abundance of *Lactobacillus* between males and females, and among the different age groups (grouped by decades) in *H. pylori* non-infected subjects. The results showed the rate of *Lactobacillus* was not affected by gender and age suggesting that the proportion of *Lactobacillus* in the gut microbiota could be associated with the severity of atrophic gastritis rather than both gender and age.

The mechanisms of the increase of *Lactobacillus* in microbiota due to a reduction of gastric acid have not been clearly elucidated. In this study, in *H. pylori*-infected subjects, the intestinal flora at species level was characterized by an increase of *L. salivarius* and a decrease of *L. acidophilus*. In *H. pylori*-infected subjects, the composition ratio of each *Lactobacillus* species was similar regardless of the severity of atrophic gastritis. Therefore, *H. pylori* infection initially influences the composition ratio of each *Lactobacillus* species in the gut microbiota before the progression of atrophic gastritis. Subsequently, the relative abundance of *Lactobacillus* increases following the decrease of gastric acid in Japanese subjects. However, in a previous German study, the intestinal flora was characterized by an increase in growth of *L. acidophilus* in *H. pylori*-infected patients (16). In that study, the microbiota was examined *via* bacterial culture, and some of *H. pylori*-infected patients had duodenal ulcers, indicating a higher gastric acid secretion. The uniqueness of the gut microbiome of Japanese might also cause the difference (15). It is necessary to examine the influence of *H. pylori* infection on the gut microbiota in different populations using next-generation analysis.

Several limitations associated with this study should be mentioned. First, we did not exclude the subjects with chronic inflammation of the gastrointestinal tract, including bacterial infection of the gut microbiota. However, prevalence rates of such diseases would be low, and the number of study subjects was sufficiently to counteract this. Second, instead of measuring gastric pH, PG I and PG I/II were used as markers of atrophic gastritis. As this study was based on a mass survey, it was not possible to measure individual gastric acid production and gastric pH. Indeed, a good correlation between serum PG levels and gastric acid secretion level has previously been shown (20). Third, we did not exclude patients with autoimmune gastritis from patients with severe atrophic gastritis. However, autoimmune gastritis is a rare disease in Japan, and the results of serum level of PGs indicated that none of the non-infected subjects had atrophic gastritis in this study. Therefore, influence of autoimmune gastritis on our results could be small.

In conclusion, the species of *Lactobacillus* in the human gut microbiota would be associated with *H. pylori* infection, and a significant increase of the rate of *Lactobacillus* appeared in subjects with severe atrophic gastritis. Efficacy of *Lactobacilli* as a probiotic might be influenced by *H. pylori* infection and subsequent atrophic gastritis, which associate with reduced gastric acid.

## Ethics Statement

This study was performed in accordance with the ethical standards of the Declaration of Helsinki and approved by the ethics committee at Hirosaki University Medical Ethics Committee (2014-377).

## Author Contributions

TS designed study, interpreted study results, and participated in drafting and editing of manuscript. CI performed statistical analysis and participated in drafting and editing of manuscript. DC assisted in study design and participated in the collection of materials. DC, TA, SN, and SF participated in the collection of materials and performed experiments.

## Conflict of Interest Statement

The authors declare that the research was conducted in the absence of any commercial or financial relationships that could be construed as a potential conflict of interest.

## References

[1] Miller LE Ouwehand AC Ibarra A. Effects of probiotic-containing products on stool frequency and intestinal transit in constipated adults: systematic review and meta-analysis of randomized controlled trials. Ann Gastroenterol (2017) 30:629–39. 10.20524/aog.2017.0192 29118557 PMC5670282

[2] Beniwal RS Arena VC Thomas L Narla S Imperiale TF Chaudhry RA A randomized trial of yogurt for prevention of antibiotic-associated diarrhea. Dig Dis Sci (2003) 48:2077–82. 10.1023/A:1021711204498 14627358

[3] Plummer S Weaver MA Harris JC Dee P Hunter J. *Clostridium* difficile pilot study: effects of probiotic supplementation on the incidence of *C. difficile* diarrhoea. Int Microbiol (2004) 7:59–62. 15179608

[4] Hickson M D’Souza AL Muthu N Rogers TR Want S Rajkumar C Use of probiotic *Lactobacillus* preparation to prevent diarrhoea associated with antibiotics: randomised double blind placebo controlled trial. BMJ (2007) 14:80. 10.1136/bmj.39231.599815.55 17604300 PMC1914504

[5] Wenus C Goll R Loken EB Biong AS Halvorsen DS Florholmen J. Prevention of antibiotic-associated diarrhoea by a fermented probiotic milk drink. Eur J Clin Nutr (2008) 62:299–301. 10.1038/sj.ejcn.1602718 17356555

[6] Imhann F Bonder MJ Vich Vila A Fu J Mujagic Z Vork L Proton pump inhibitors affect the gut microbiome. Gut (2016) 65:740–8. 10.1136/gutjnl-2015-310376 26657899 PMC4853569

[7] Jackson MA Goodrich JK Maxan ME Freedberg DE Abrams JA Poole AC Proton pump inhibitors alter the composition of the gut microbiota. Gut (2016) 65:749–56. 10.1136/gutjnl-2015-310861 26719299 PMC4853574

[8] Takashima M Furuta T Hanai H Sugimura H Kaneko E. Effects of *Helicobacter pylori* infection on gastric acid secretion and serum gastrin levels in Mongolian gerbils. Gut (2001) 48:765–73. 10.1136/gut.48.6.765 11358893 PMC1728329

[9] Hammond CE Beeson C Suarez G Peek RM Jr Backert S Smolka AJ. *Helicobacter pylori* virulence factors affecting gastric proton pump expression and acid secretion. Am J Physiol Gastrointest Liver Physiol (2015) 309:G193–201. 10.1152/ajpgi.00099.2015 26045613 PMC4525105

[10] Kuipers EJ Uyterlinde AM Peña AS Roosendaal R Pals G Nelis GF Long-term sequelae of *Helicobacter pylori* gastritis. Lancet (1995) 17:1525–8. 10.1016/S0140-6736(95)91084-0 7791437

[11] Weck MN Gao L Brenner H. *Helicobacter pylori* infection and chronic atrophic gastritis: associations according to severity of disease. Epidemiology (2009) 20:569–74. 10.1097/EDE.0b013e3181a3d5f4 19404195

[12] Kishikawa H Nishida J Ichikawa H Kaida S Takarabe S Matsumkubo T Fasting gastric pH of Japanese subjects stratified by IgG concentration against *Helicobacter pylori* and pepsinogen status. Helicobacter (2011) 16:427–33. 10.1111/j.1523-5378.2011.00868.x 22059393

[13] Shimoyama T Aoki M Sasaki Y Matsuzaka M Nakaji S Fukuda S. ABC screening for gastric cancer is not applicable in a Japanese population with high prevalence of atrophic gastritis. Gastric Cancer (2012) 15:331–4. 10.1007/s10120-012-0141-x 22282137

[14] Ishimura N Owada Y Aimi M Oshima T Kamada T Inoue K No increase in gastric acid secretion in healthy Japanese over the past two decades. J Gastroenterol (2015) 50:844–52. 10.1007/s00535-014-1027-y 25501288

[15] Nishijima S Suda W Oshima K Kim SW Hirose Y Morita H The gut microbiome of healthy Japanese and its microbial and functional uniqueness. DNA Res (2016) 23(2):125–33. 10.1093/dnares/dsw002 26951067 PMC4833420

[16] Bühling A Radun D Müller WA Malfertheiner P. Influence of anti-*Helicobacter* triple-therapy with metronidazole, omeprazole and clarithromycin on intestinal microflora. Aliment Pharmacol Ther (2001) 15:1445–52. 10.1046/j.1365-2036.2001.01033.x 11552917

[17] Kawai T Kawakami K Kudo T Ogiahara S Handa Y Moriyasu F. A new serum antibody test kit (E plate) for evaluation of *Helicobacter pylori* eradication. Intern Med (2002) 41:780–3. 10.2169/internalmedicine.41.780 12412995

[18] Sato M Shimoyama T Takahashi R Kajiyama H Sano Y Sakaedani N Characterization and usefulness of stool antigen tests using a monoclonal antibody to *Helicobacter pylori* catalase. J Gastroenterol Hepatol (2012) 27:23–8. 10.1111/j.1440-1746.2012.07066.x 22486867

[19] Miki K Ichinose M Kawamura N Matsushima M Ahmad HB Kimura M The significance of low serum pepsinogen levels to detect stomach cancer associated with extensive chronic gastritis in Japanese subjects. Jpn J Cancer Res (1989) 80:111–4. 10.1111/j.1349-7006.1989.tb02276.x 2498245 PMC5917707

[20] Miki K. Gastric cancer screening using the serum pepsinogen test method. Gastric Cancer (2006) 9:245–53. 10.1007/s10120-006-0397-0 17235625

[21] Takahashi S Tomita J Nishioka K Hisada T Nishijima M. Development of a prokaryotic universal primer for simultaneous analysis of bacteria and *Archaea* using next-generation sequencing. PLoS One (2014) 9:e105592. 10.1371/journal.pone.0105592 25144201 PMC4140814

[22] Hisada T Endoh K Kuriki K. Inter- and intra-individual variations in seasonal and daily stabilities of the human gut microbiota in Japanese. Arch Microbiol (2015) 197:919–34. 10.1007/s00203-015-1125-0 26068535 PMC4536265

[23] Iijima K Koike T Abe Y Shimosegawa T. Cutoff serum pepsinogen values for predicting gastric acid secretion status. Tohoku J Exp Med (2014) 232:293–300. 10.1620/tjem.232.293 24717778

